# Hemodynamic changes in the portal vein with age: evaluation using four-dimensional flow MRI

**DOI:** 10.1038/s41598-023-34522-z

**Published:** 2023-05-06

**Authors:** Chung-Man Moon, Seul-Kee Kim, Suk‑Hee Heo, Sang-Soo Shin

**Affiliations:** 1 Research Institute of Medical Sciences, Chonnam National University, Gwangju, Republic of Korea; 2 Department of Radiology, Chonnam National University Hwasun Hospital, Hwasun, Republic of Korea; 3 Department of Radiology, Chonnam National University Hospital, Gwangju, Republic of Korea; 4 Department of Radiology, Chonnam National University Medical School, 42 Jebong-ro, Dong-gu, Gwangju, 61469 Republic of Korea

**Keywords:** Magnetic resonance imaging, Ageing

## Abstract

Aging process is associated with gradual change of liver function and structure. The goal of this study was to evaluate age-related hemodynamic changes in the portal vein (PV) using four-dimensional (4D) flow MRI in healthy adults. A total of 120 healthy subjects were enrolled and categorized into groups A (n = 25, 30–39 years), B (n = 31, 40–49 years), C (n = 34, 50–59 years), and D (n = 30, 60–69 years). All subjects underwent 4D flow data acquisition using a 3-T MRI system to measure the hemodynamic parameters in the main PV. The clinical characteristics and 4D flow parameters were compared among the groups using analysis of variance and analysis of covariance after controlling for significant covariates, accordingly. The outcome metric applying the age-related quadratic model to estimate the age at which 4D flow parameters are the highest (the peak age) as well as the rates of age-related 4D flow changes was estimated. The average area, average through-plane velocity, peak velocity magnitude, average net flow, peak flow, and net forward volume in group D were significantly lower than those in groups A, B and C (*P* < 0.05). Group C showed significantly lower values of the average through-plane velocity and peak velocity magnitude than those of group B (*P* < 0.05). The peak age computed was approximately 43–44 years of age for all 4D flow parameters. The rates of age-related 4D flow changes for all 4D flow parameters were negatively correlated with age (*P* < 0.05). The volume and velocity of the blood flow through the PV peaked at approximately 43–44 years of age and decreased significantly after 60 years of age.

## Introduction

The hepatic hemodynamic status is important to understand the pathophysiological mechanism and progression of the liver diseases^1^. In particular, the portal vein (PV) is the main vessel of the liver blood system and delivers approximately 75% of the blood supply by draining blood from the splenic and superior mesenteric veins to the liver to metabolize nutrients and remove potentially toxic substances^2^. The normal flow waveform in the PV shows constant, non-pulsatile, and unidirectional blood flow toward the liver^2^. Indeed, any changes in the blood flow pattern in the PV might be characteristic features of various pathologies including portal hypertension in liver cirrhosis^3^. Therefore, changes in the volume and velocity of the blood flow through the PV with age may have important implications for the health of the liver.

The process of aging is associated with gradual deterioration of hepatic function and structure accompanied by various changes in liver cells such as sinusoidal endothelial cells^4^. More seriously, aging can also increase mortality risk from various liver diseases as an adverse prognostic factor^5^. To date, although a few studies^6–8^ including a small number of subjects have used ultrasound to measure portal blood flow and shed light on the effect of the age on the hemodynamic circumstances in the PV, the hemodynamic characteristics of the PV including the volume and velocity of the blood flow through the PV with age remain poorly understood. The studies^6,8^ published in 1989 have reported that liver volume and portal blood flow decrease after the age of 50 years, and liver function decreases progressively with aging. Another study^7^ revealed that total hepatic flow measured by pulsed echo-Doppler significantly decreased with age, particularly in subjects over 75 years. A similar reduction was also observed in functional hepatic flow, and both were correlated with age. Accordingly, when reliably evaluating altered hepatic blood flow associated with liver diseases, understanding age-related hemodynamic changes in the PV as a baseline could be important. Also, ultrasonography has been mainly used to assess changes in portal and splanchnic blood flow to the liver. However, it is sometimes limited for visualizing blood flow due to overlying intestinal gas and a limited field of view. Furthermore, this modality could lead to inaccurate quantification of hemodynamics as a result of variability in the actual velocity values of the portal venous blood flow^9^.

Four-dimensional (4D) flow magnetic resonance imaging (MRI) has recently attracted attention as a noninvasive imaging tool to provide relevant hemodynamic and anatomic information for clinical diagnosis of various abdominal pathologies^10^. There was a clinical study^11^ using 4D flow MRI that investigated normal three-dimensional portal venous hemodynamics in healthy subjects in their 20 s and 50 s and in patients with liver cirrhosis. Nevertheless, the correlation of blood flow characteristics with age in the portal venous system has not been well characterized in healthy subjects.

Therefore, this study aimed to investigate hemodynamic changes in the PV with age using 4D flow MRI in healthy adults.

## Results

### Clinical characteristics

Table 1 presents the demographics and clinical characteristics of the four groups. There were significant differences in the age (*P* < 0.001), body weight (*P* = 0.007), body height (*P* < 0.001), and standard liver volume (*P* < 0.001) among the groups. Further post hoc test revealed that there was a significantly different at *P* < 0.05 level between the groups, as detailed in Table 1. However, there were no significant differences in other variables among the groups.

**Table 1:** Comparison of demographics and biochemical serum parameters among the four groups.

Variables	Group A	Group B	Group C	Group D	*P*-value^a^					
	(n = 25)	(n = 31)	(n = 34)	(n = 30)	P1	P2	P3	P4	P5	P6
Age (years)	33.60 ± 2.06	43.87 ± 3.06	58.18 ± 2.69	65.87 ± 3.06	0.00	0.00	0.00	0.00	0.00	0.00
Sex (male/female)	12/13	13/18	19/15	16/14	0.69^b^					
Body weight (kg)	63.96 ± 6.72	64.32 ± 10.80	61.62 ± 10.26	56.60 ± 8.76	1.00	0.83	0.04	0.72	0.02	0.22
Body height (cm)	169.84 ± 7.59	165.42 ± 8.83	164.41 ± 7.97	158.33 ± 8.73	0.28	0.11	0.00	0.97	0.01	0.04
Body mass index (kg/m^2^)	22.20 ± 2.10	23.55 ± 3.76	22.70 ± 2.62	22.57 ± 3.05	0.41	0.94	0.97	0.72	0.65	1.00
Standard liver volume (cm^3^)	1227.98 ± 81.33	1213.15 ± 113.43	1184.54 ± 118.53	1114.28 ± 105.80	0.97	0.50	0.00	0.76	0.01	0.08
Spleen diameter (cm)	9.90 ± 0.80	9.87 ± 0.86	9.89 ± 1.37	9.77 ± 1.27	1.00	1.00	0.98	1.00	0.99	0.98
Platelet count (× 1000/µL)	249.36 ± 93.51	240.77 ± 73.07	260.47 ± 100.77	236.27 ± 71.82	0.99	0.97	0.96	0.84	1.00	0.74
Aspartate aminotransferase (U/L)	20.17 ± 7.28	22.33 ± 7.05	25.64 ± 13.19	25.15 ± 8.60	0.92	0.29	0.54	0.75	0.89	1.00
Alanine aminotransferase (U/L)	21.26 ± 6.66	19.28 ± 8.59	23.80 ± 13.60	21.92 ± 7.88	0.94	0.85	1.00	0.54	0.91	0.96
Lactate dehydrogenase (U/L)	351.57 ± 89.45	400.94 ± 99.39	390.70 ± 113.74	426.62 ± 48.78	0.48	0.62	0.17	0.99	0.91	0.77

P1, Group A versus Group B; P2, Group A versus Group C; P3, Group A versus Group D; P4, Group B versus Group C; P5, Group B versus Group D; P6, Group C versus Group D.

^a^One-way analysis of variance (ANOVA) with Scheffe's post hoc test among the groups.

^b^Chi-square test.

### Comparison of the quantified parameters from 4D flow MRI among the four groups

**Figure 1:**
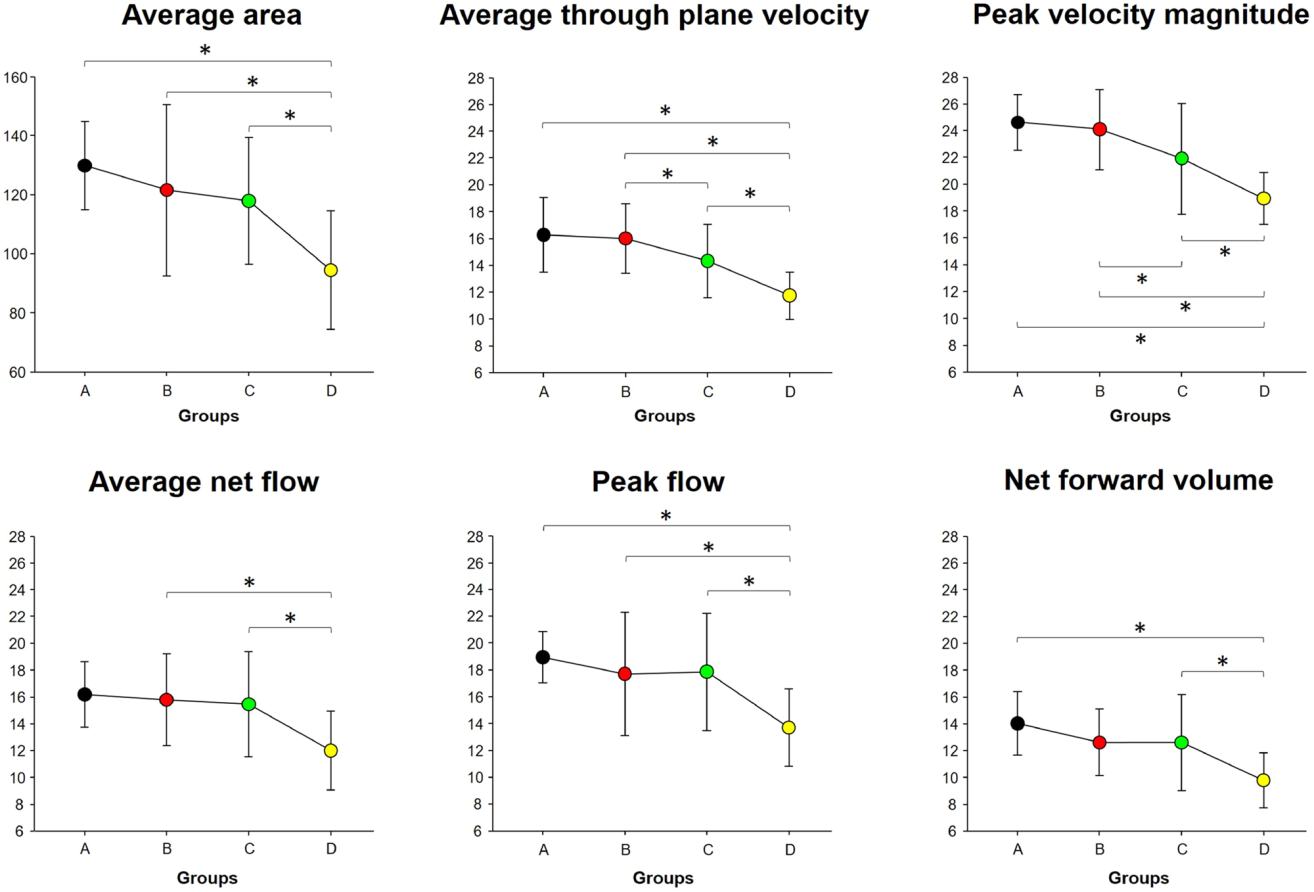
Comparison of hemodynamic parameters among the four groups using analysis of covariance (ANCOVA) with adjustments for body weight, body height, and standard liver volume. *Significant difference with post-hoc test at *P* < 0.05.

As shown in Fig. 1, in group D, all hemodynamic parameters in the main PV using 4D flow MRI including the average area, average through-plane velocity, peak velocity magnitude, average net flow, peak flow, and net forward volume were significantly lower than those in groups A, B, and C (except for the average net flow in group A and net forward volume in group B) (*P* < 0.05). Moreover, group C showed significantly lower values of the average through-plane velocity and peak velocity magnitude than those in group B (*P* < 0.05). However, there were no significant differences in other 4D flow parameters among the groups.

The average intraclass correlation coefficients (ICC) for all 4D flow parameters in the PV were 0.911 ± 0.043 for group A; 0.890 ± 0.070 for group B; 0.912 ± 0.053 for group C; and 0.936 ± 0.023 for group D (Table 2). All *P*-values were less than 0.001 for ICC tests.

**Table 2:** Interobserver variability in 4D flow parameters.

	ICC (95% CI)*
Group A	
Average area (mm^2^)	0.886 (0.758–0.948)
Average through-plane velocity (cm/sec)	0.953 (0.896–0.979)
Peak velocity magnitude (cm/sec)	0.844 (0.679–0.928)
Average net flow (mL/sec)	0.938 (0.865–0.972)
Peak flow (mL/sec)	0.896 (0.779–0.953)
Net forward volume (mL)	0.948 (0.885–0.977)
Group B	
Average area (mm^2^)	0.900 (0.803–0.950)
Average through-plane velocity (cm/sec)	0.788 (0.560–0.898)
Peak velocity magnitude (cm/sec)	0.821 (0.662–0.910)
Average net flow (mL/sec)	0.928 (0.857–0.965)
Peak flow (mL/sec)	0.946 (0.890–0.973)
Net forward volume (mL)	0.959 (0.916–0.980)
Group C	
Average area (mm^2^)	0.883 (0.779–0.940)
Average through-plane velocity (cm/sec)	0.886 (0.785–0.942)
Peak velocity magnitude (cm/sec)	0.833 (0.692–0.913)
Average net flow (mL/sec)	0.948 (0.898–0.974)
Peak flow (mL/sec)	0.951 (0.904–0.975)
Net forward volume (mL)	0.971 (0.943–0.985)
Group D	
Average area (mm^2^)	0.946 (0.890–0.974)
Average through-plane velocity (cm/sec)	0.908 (0.816–0.955)
Peak velocity magnitude (cm/sec)	0.907 (0.814–0.955)
Average net flow (mL/sec)	0.945 (0.889–0.974)
Peak flow (mL/sec)	0.946 (0.890–0.975)
Net forward volume (mL)	0.962 (0.921–0.982)

*ICC* Intraclass correlation coefficient, *CI* Confidence interval.

The *P*-values of ICC were statistically significant (all *P* < 0.001).

*The interobserver reliability between the two observers was analyzed by the ICC test.

### Correlation of the 4D Flow parameters with clinical characteristics in all age groups

Among various clinical characteristics, body weight (*P* = 0.007), body height (*P* < 0.001), standard liver volume (*P* < 0.001), and age (*P* < 0.001) had significant *P*-values among the four groups (Table 1). In Table 3, regarding Pearson’s correlation outcomes, the 4D flow MRI parameters, including the average through-plane velocity, peak velocity magnitude, average net flow, peak flow, and net forward volume in the PV, were positively correlated with body weight, body height, and standard liver volume (*P* < 0.05). In contrast, age was negatively correlated with these parameters (*P* < 0.05).

**Table 3:** Correlation coefficients between the levels of 4D flow parameters and clinical characteristics in all groups.

Parameters	Average area (mm^2^)	Average through-plane velocity (cm/sec)	Peak velocity magnitude (cm/sec)	Average net flow (mL/sec)	Peak flow (mL/sec)	Net forward volume (mL)
Pearson’s correlation						
Age (years)	− 0.484	− 0.552	− 0.554	− 0.415	− 0.437	− 0.481
Body weight (kg)	0.496	0.439	0.452	0.541	0.387	0.558
Body height (cm)	0.702	0.637	0.489	0.541	0.466	0.615
Standard liver volume (cm^3^)	0.613	0.546	0.513	0.601	0.456	0.636
Partial correlation*						
Age (years)	− 0.216	− 0.354	− 0.405	− 0.185	− 0.254	− 0.247

All values are significant at *P* < 0.05.

*A partial correlation coefficient between 4D flow parameters and age was calculated after controlling for body weight, body height, and standard liver volume.

In the partial correlation outcomes, all 4D flow parameters were negatively correlated with age, even after adjusting for other factors including body weight, body height, and standard liver volume (*P* < 0.05).

### Estimation of the peak age and age-related 4D flow change rates

**Figure 2:**
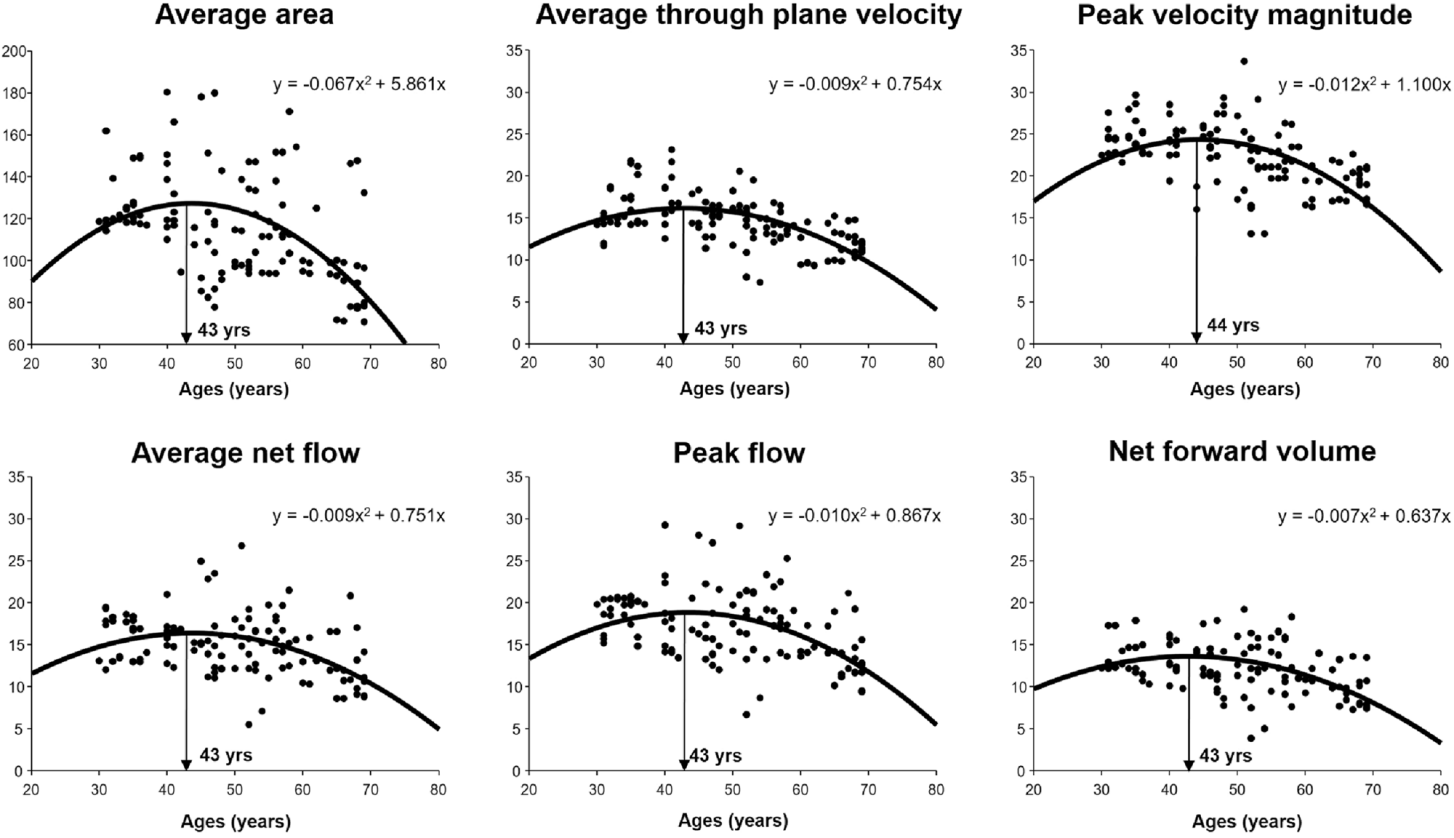
Age trajectories of 4D flow parameters for all groups with the peak age marks (arrows).

**Figure 3:**
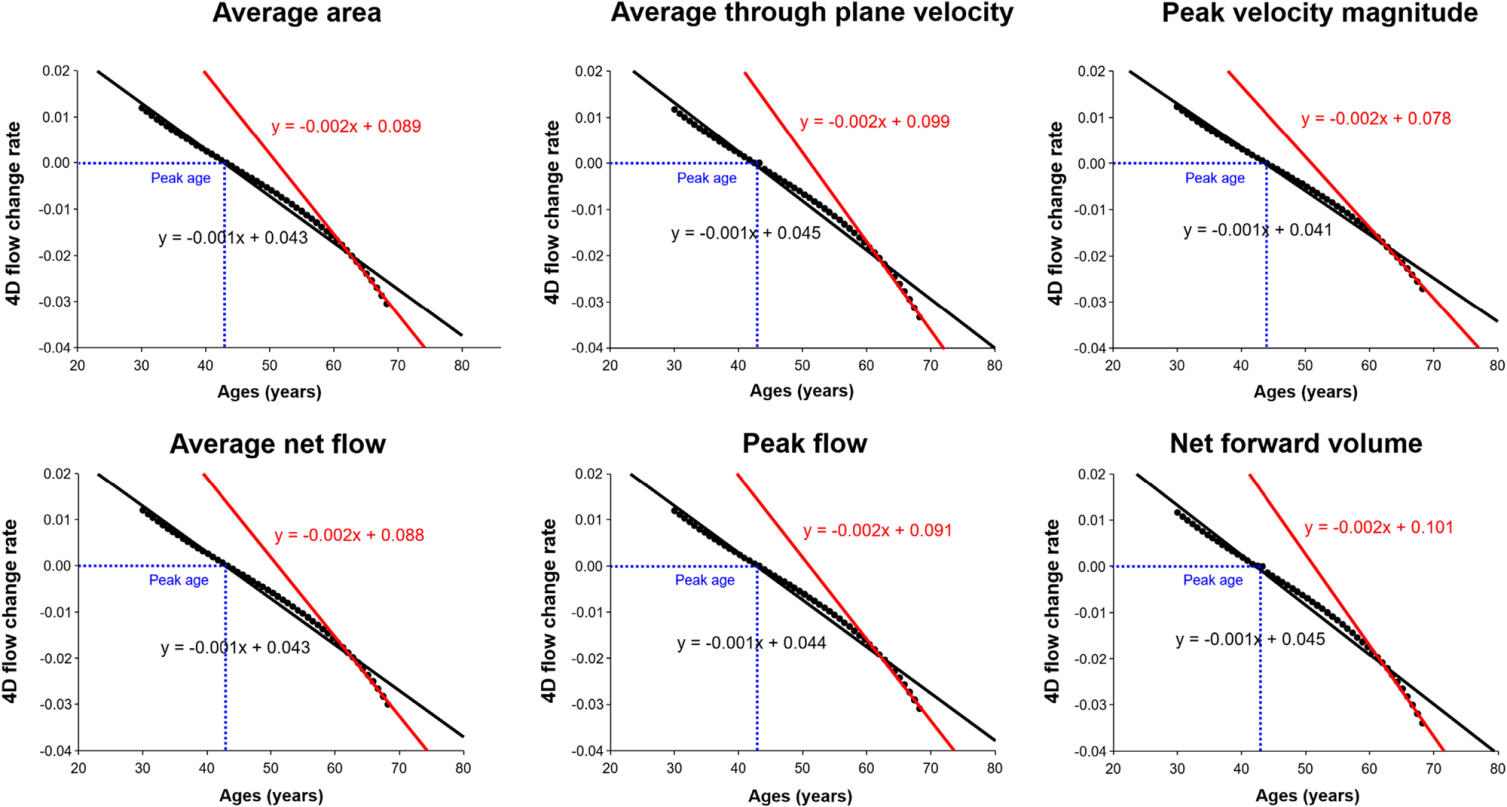
Correlation between the rates of change in age-related 4D flow parameters obtained from a quadratic model of the peak age and ages in all groups (black line) and in group D (red line). The blue line indicates the point of the peak age.

Figure 2 shows the computation of the peak age for 4D flow parameters using the quadratic model. The peak age computed was 43 years of age for the average area, 43 years of age for the average through-plane velocity, 44 years of age for the peak velocity magnitude, 43 years of age for the average net flow, 43 years of age for the peak flow, and 43 years of age for the net forward volume. As shown in Fig. 3, for all parameters, the rates of age-related 4D flow change were negatively correlated with age (*P* < 0.05) in all groups (y-intercept: 0.041–0.045, slope: − 0.001), group A (y-intercept: 0.040, slope: − 0.001), group B (y-intercept: 0.036–0.037, slope: − 0.001), group C (y-intercept: 0.043–0.049, slope: − 0.001), and group D (y-intercept: 0.078–0.101, slope: − 0.002). The age-related 4D flow change rate observed before the peak age tended to decrease gradually, whereas this trend in the change rate gradually increased after the peak age with aging. In particular, the y-intercept and slope were higher in group D than in the other groups.

## Discussion

In an effort to overcome the limitations when using ultrasonography as described in the introduction section, our study investigated the age-related hemodynamic changes in the main PV in a cohort of more than 100 healthy subjects using a 4D flow MRI, which has been shown to make reproducible flow measurements through comprehensive dataset of anatomical and functional flow information^2^. In fact, in our study, the range of ICC values, as an indicator of reproducibility of the measured values, was 0.89–0.93, suggesting that the reliability of 4D flow MRI is very good.

This study was consistent with the trend of a previous study^7^ that enrolled 40 normal subjects in four age groups (< 45, 45–60, 61–75 and > 75 years). That study, published in 1999, found that total hepatic blood flow (the sum of portal and hepatic arterial blood flow) and functional hepatic blood flow decreased significantly with age^7^. Our findings showed that the values of blood flow parameters in the main PV decreased significantly after 60 years of age. In our study, all portal blood flow parameters were significantly lower in normal subjects older than 60 years of age than in those younger than 40 years of age. Namely, the average area, average through plane velocity, peak velocity magnitude, average net flow, peak flow, and net forward volume decreased by 27%, 27%, 23%, 25%, 27%, and 30%, respectively. Furthermore, the temporal trajectories of 4D flow parameters of the PV were mapped in healthy subjects covering an adult lifespan of more than 40 years of age. As a result, the hemodynamic parameters of the PV peaked at approximately 43–44 years of age.

Zoli et al.^7^ suggested that age-related reductions in liver weight and volume might be explained by a decrease in the number and size of hepatocytes. Another ultrasound study^8^ showed a 20–40% decrease in liver volume with age. In fact, the volume of liver cells gradually increases as they mature but begins to decrease with aging^12^. An experimental study^13^ reported that aging-related changes in liver cells included polyploidy, accumulation of lipofuscin inside hepatocytes, decreased smooth endoplasmic reticulum area, and decreased mitochondrial number and functional decline. With aging, hepatocyte polyploidy tends to occur more frequently, accompanied by a decrease in the number of mitochondria and dysfunction, resulting in decreased adenosine triphosphate levels^12^. A molecular imaging study^14^ using ^99m^Tc-galactosyl-human serum albumin liver scintigraphy reported a decrease in functional liver cell mass but not total liver volume associated with aging-related changes in the liver. In addition, some studies have suggested that aging causes severe morphological changes in the sinusoidal vasculature, negatively affecting liver function^15^. Taken together, we hypothesized that aging, defined as the gradual changes in liver structure and function that occurs over time and various changes in hepatocytes, could eventually lead to hemodynamic changes in the PV. Following this hypothesis, our data showed a positive correlation of body weight, height, and standard liver volume with 4D flow MRI parameters. Furthermore, our data demonstrated that aging itself is an important factor in reducing portal blood flow, even if the influence of variables affecting portal blood hemodynamics such as liver volume, height, and weight were excluded.

Our novel study using 4D flow MRI provided a new metric for assessing age-dependent portal blood flow characteristics in healthy subjects, implying the importance of an age-matched control cohort for assessing liver disease associated with PV blood flow. Interestingly, this study revealed for the first time that the age at which the hemodynamic parameters of PV reach the highest values (maximum age) was the mid-40 s. At the same time, the trend in the rate of change of flow hemodynamics according to age gradually increased after the peak observation; in particular, it increased more rapidly in the 60 s. This may be due to long-term risk factors such as increased oxidative stress, increased inflammatory responses, accelerated cellular aging, and progressive organ dysfunction with aging^16^. Similarly, only a small number of studies published 30 years ago^6,8^ suggested that portal blood flow decreased after age 50 and postulated that aging-related decreased blood flow may be an important component of age-related changes in the liver. These changes may negatively affect the process by which various drugs are eliminated from the body with aging. Therefore, the peak age and age-related rate of change in 4D flow values are considered important metrics for understanding the physiological and/or pathological course of liver disease with age. However, it should be noted that age is closely related to other basic metrics including weight, height, and liver volume. These key factors must be considered together when assessing the diagnosis and outcome of various liver diseases^17^.

There are several limitations in this study. First, because our study was conducted at a single tertiary care medical center, the results may not be representative of the entire population. Large-scale studies with populations that reflect different traits may help to better validate the results. Second, the metrics presented in this study were the results of a cross-sectional study. This potentially limits the conclusions that can be drawn about the longitudinal process of aging. Therefore, further studies with a longitudinal design are needed to confirm the cross-sectional trends observed here. Third, the age at which the 4D flow parameters reach the highest values (peak age) is considered a biologically meaningful indicator, but the clinical significance of this indicator requires further studies.

In conclusion, our study demonstrates age-related hemodynamic changes in the PV using 4D flow MRI in healthy subjects, where the volume and velocity of the blood flow through the PV peaked at approximately 43–44 years of age and decreased significantly after 60 years of age.

## Methods

### Study population

**Figure 4:**
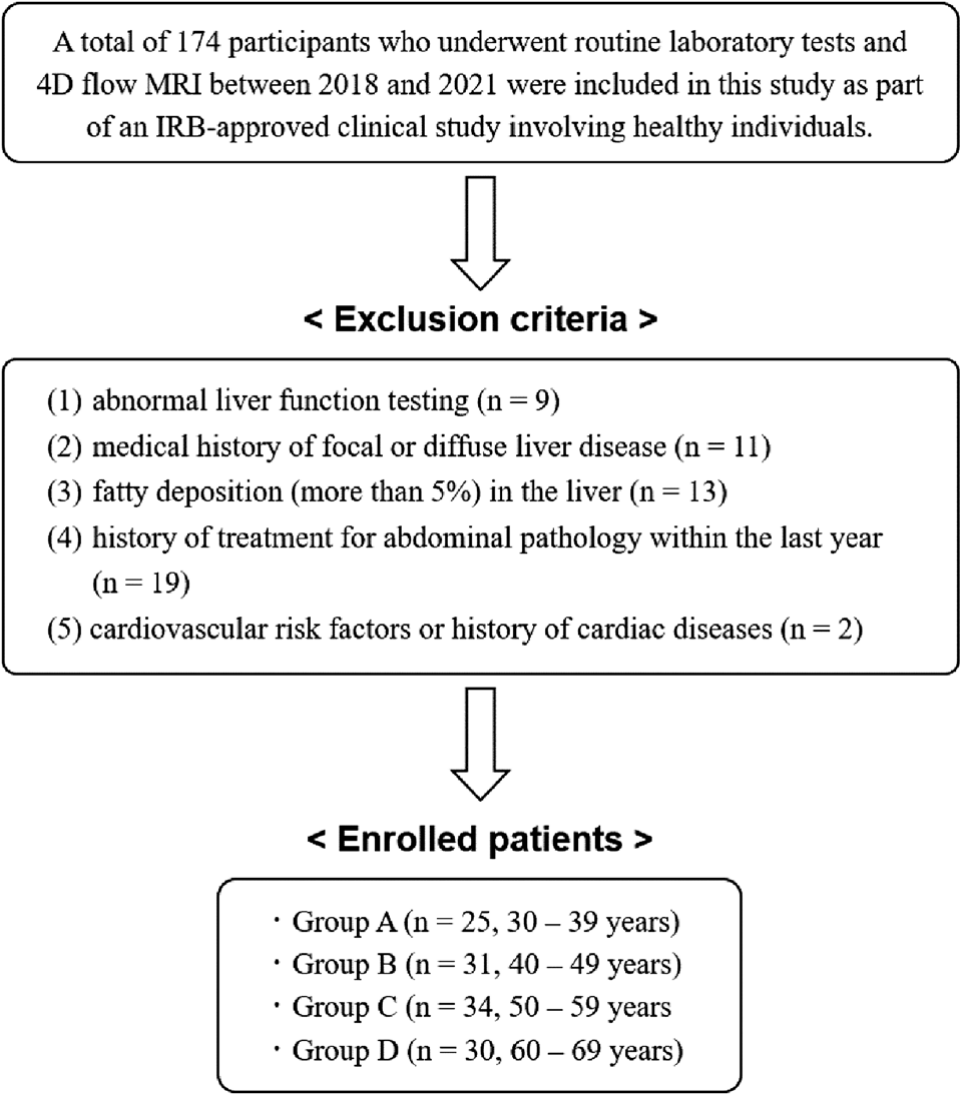
Flow diagram of enrollment and exclusion from participant cohorts.

At least 25 subjects for each group were estimated to significantly quantify the differences in 4D flow parameters among the four groups with an α error of 0.05 and a β error of 0.2. A total of 174 participants who underwent routine laboratory tests including liver function testing and 4D flow MRI between 2018 and 2021 were included in this study as part of an IRB-approved clinical study involving healthy individuals which was approved by the institutional review board of Chonnam National University Hospital, and conformed to the ethical guidelines of the 2008 Declaration of Helsinki. Written informed consent was obtained from all subjects. And the methods in this study were carried out in accordance with the approved guideline and regulation. Of those participants, 54 subjects were excluded from our study due to the following reasons: (1) abnormal liver function testing (n = 9); (2) medical history of focal or diffuse liver disease (n = 11); (3) fatty deposition (more than 5%) in the liver (n = 13); (4) history of treatment for abdominal pathology within the last year (n = 19); and (5) cardiovascular risk factors or history of cardiac diseases (n = 2). Finally, 120 consecutive individuals were enrolled in this study. Each subject was categorized into one of four age groups: group A (n = 25, 30–39 years); group B (n = 31, 40–49 years); C (n = 34, 50–59 years); and D (n = 30, 60–69 years) (Fig. 4).

### 4D flow MRI acquisition

**Figure 5:**
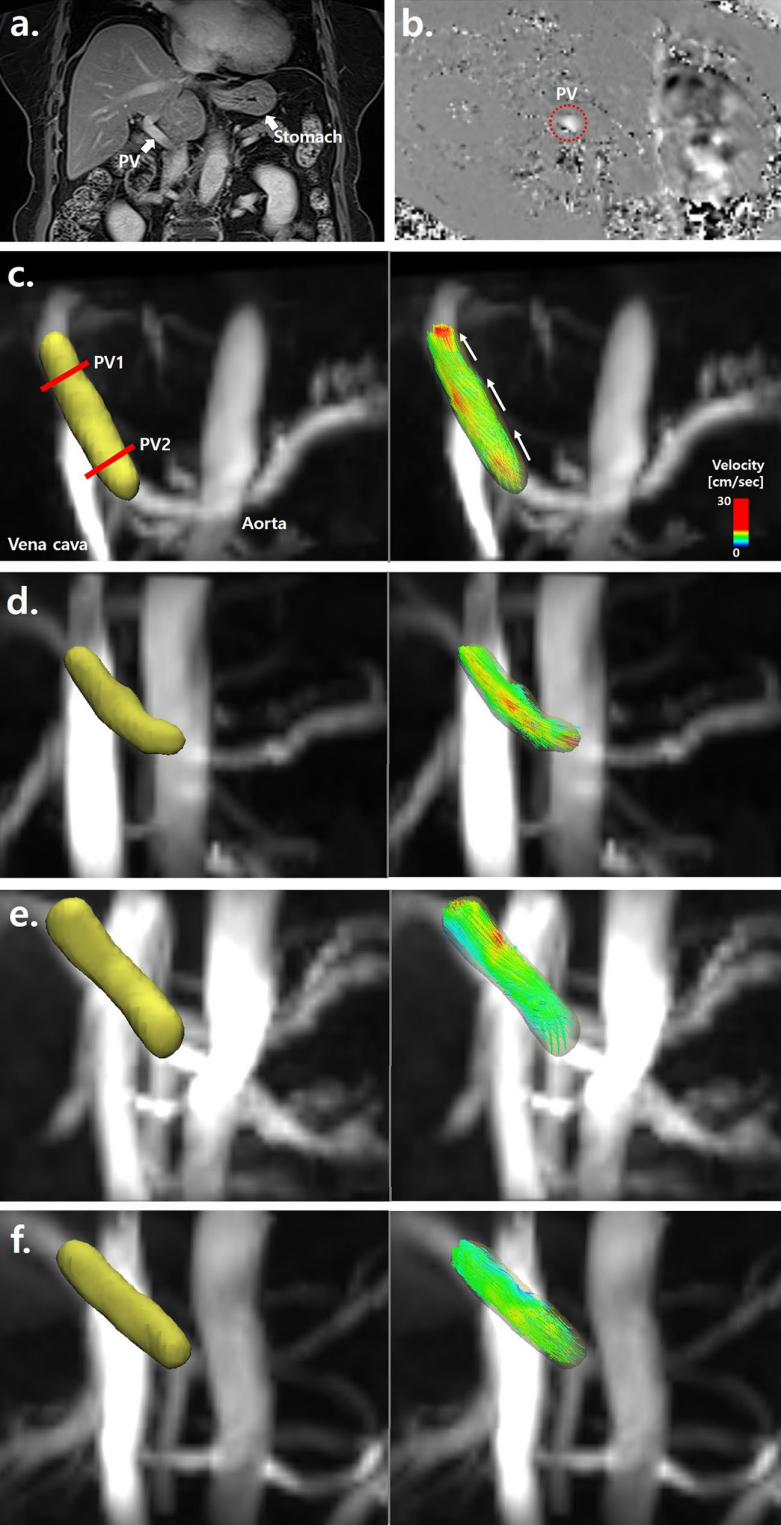
Anatomic coronal MR image (**a**) in the abdomen showing the main portal vein (PV) and the status of the stomach. Velocity-encoding (VENC) image (**b**) focusing on the PV (VENC = 30 cm/s). The red dotted circle indicates the cross-sectional area of the PV. Four-dimensional flow MRI data were post-processed; then, segmented anatomic images (left) and color-coded streamline images (right) were generated from a 38-year-old male (**c**), a 45-year-old female (**d**), a 53-year-old male (**e**), and a 68-year-old female (**f**). The red solid lines in (**c**) indicate planes for measuring the hemodynamic parameters, and the white arrows in (**c**) show the direction of blood flow.

All subjects underwent 4D flow MRI using a clinical 3-T scanner (Skyra; Siemens Healthcare, Erlangen, Germany) with a 32-channel phased array coil. The 4D flow sequence covered the main PV (Fig. 5) and utilized both ECG-gated and navigator-triggering techniques. The imaging parameters for 4D flow MRI were as follows: repetition time/echo time = 6.72/2.84 ms, field-of-view = 380 × 308 mm^2^, slice thickness = 2.0 mm, matrix = 192 × 129, GRAPPA acceleration factor = 3, Cartesian sampling of k-space, number of excitations = 1, and scan time = 8–15 min. Four-dimensional flow MRI scans were performed after a period of fasting for at least 8 h, and the status of the stomach was reviewed on anatomic MR images (Fig. 5a). To avoid velocity aliasing, the velocity encoding was performed prior to the 4D flow MRI. Through the velocity-encoding sensitivity [VENC] scout images (Fig. 5b), the VENC was set to 30 cm/s^18^.

### 4D flow MRI data analysis

Post-processing of 4D flow images was performed using Siemens 4D flow software (V2.4, Siemens Healthcare, Erlangen, Germany) and quantified by two radiologists with eight and 20 years of experience in interpreting abdominal MRIs, respectively; the 4D flow parameters measured by two observers were averaged to set as the 4D flow parameter values. Time-resolved images of the 3D velocity vector fields were generated to display the blood flow in the PV. Thereafter, 3D streamlines and time-density curves of the blood flow parameters were obtained from the 4D datasets. The quantitative parameters included the average area (mm^2^), average through-plane velocity (cm/sec), peak velocity magnitude (cm/sec), average net flow (mL/sec), peak flow (mL/sec), and net forward volume (mL) in two planes of the PV. The 4D flow parameters obtained from two cut planes were averaged to represent the values of main PV.

### Clinical data acquisition

Various clinical information, including age, sex, body weight, body height, body mass index, standard liver volume, splenic diameter, platelet count, and levels of aspartate aminotransferase, alanine aminotransferase, and lactate dehydrogenase, were obtained through electronic chart analysis. Blood samples were analyzed using a routine clinical chemistry analyzer to measure blood cell counts and liver function. The splenic diameter was measured on MRI using electronic calipers and was defined as the greatest longitudinal dimension at the level of the splenic hilum on the PACS monitor^19^. Furthermore, the formulas for standard liver volume identified in the literature were used to calculate standard liver volume in each subject, and simple subject-specific parameters such as weight and height were also used^20^.

### Statistical analysis

Data were analyzed using SPSS 24.0 software (SPSS Inc., Chicago, IL). Clinical characteristics were compared using analysis of variance (ANOVA), and 4D flow measurements were compared using analysis of covariance (ANCOVA) with body weight, body height, and standard liver volume as confounding factors in the test as covariates among the four groups. The ICC was used to assess the interobserver agreement in quantitative measurements of 4D flow parameters determined by two observers. Pearson’s correlation analysis was applied to analyze the relationship between the 4D flow parameters and clinical characteristics in all subjects, and partial correlation was performed to evaluate the relationship between the 4D flow parameters and age after controlling for body weight, body height, and standard liver volume. In addition, the outcome metric applying age-related quadratic model to estimate the age at which 4D flow parameters are the highest (the peak age) was evaluated. The rates of age-related 4D flow changes before and after the peak age were estimated by the following formula: (y2 − y1)/y1, where y2 and y1 were obtained from the age-related quadratic model for the peak age, representing the change extent in 4D flow values with age. Then, Pearson’s correlation analysis was performed using these values from all groups as well as from each group to compare the slope and intercept between those from all groups and each group.

## Data Availability

The data that support the findings of this study are available from the corresponding author upon reasonable request.
